# Haematological Manifestations of SARS-CoV-2: Insights into Erythropoiesis, Hepcidin Regulation, and Cytokine Storm

**DOI:** 10.3390/ijms26030874

**Published:** 2025-01-21

**Authors:** Elahi Parham, Makky Ahmad, Marco Falasca

**Affiliations:** 1 Department of Medicine and Surgery, University of Parma, 43125 Parma, Italy; parham.elahi@studenti.unipr.it; 2 Department of Medicine and Surgery, University of Pavia, 27100 Pavia, Italy; ahmad.makky01@universitadipavia.it

**Keywords:** COVID-19, SARS-CoV-2, erythropoiesis, haematology, hepcidin, interleukin, circulating CD71+ erythroid cells, hypoxia

## Abstract

Severe acute respiratory syndrome coronavirus 2 (SARS-CoV-2) causes COVID-19, a respiratory disease that can range in presentation from mild symptoms to severe conditions such as pneumonia and acute respiratory distress syndrome. SARS-CoV-2, a single-stranded RNA virus, spreads through aerosols and respiratory droplets. It enters human cells by binding to the angiotensin-converting enzyme 2 receptor, leading to various complications, including significant alterations in red blood cells and potential disruptions in haemoglobin function and oxygen transport. During infection, the interaction between hypoxia, inflammation, and haematopoiesis affects erythropoiesis at multiple levels. Hypoxia and inflammation, resulting from lung complications and a reduced red blood cell count, influence the regulation of hepcidin, a key regulator of iron levels in the blood. Elevated hepcidin levels are associated with hypoxia and the suppression of erythroferrone, a hormone that normally inhibits hepcidin production. Despite high levels of inflammation, patients in intensive care units often exhibit elevated ferritin levels, which, rather than indicating low hepcidin, suggest disrupted iron metabolism and the development of severe anaemia. Iron is kept in stores, likely due to paradoxically high hepcidin levels, which explains the elevated ferritin measurements. An increase in immature blood cells and a decrease in CD71+ erythroid cells are observed. The elevated levels of CD71+ erythroid cells highlight their dual role in modulating hyper-inflammation and immune response during disease progression. This review examines the pathway by which SARS-CoV-2 affects red blood cell production and the haematopoietic system and how it triggers cytokine storms through interleukins, immature blood cells, and CD71+ erythroid cells. Understanding these processes provides novel pathways for managing haematological manifestations and immune responses in patients with COVID-19.

## 1. Introduction

Coronavirus disease 2019 (COVID-19), caused by severe acute respiratory syndrome coronavirus 2 (SARS-CoV-2), has numerous pathophysiological effects, particularly impacting the upper and lower respiratory tracts [1,2,3]. In addition, this virus can chronically disrupt erythropoiesis and iron metabolism due to its pro-inflammatory and hypoxic effects [4]. COVID-19 can also trigger a cytokine storm, known as cytokine release syndrome (CRS) [5,6,7], which is characterised by intense and disorganised hyper-inflammation generated by the immune system during infections [6,7]. The connection between COVID-19 and CRS is thought to operate through two main mechanisms: viral replication and the interaction between antibodies and antigens [8]. In the first mechanism, viral replication triggers pyroptosis, with IFN-α playing a role in the initial antiviral response, while IFN-γ drives the subsequent inflammatory cascade [9,10]. Pyroptosis is a form of inflammatory programmed cell death that, unlike apoptosis, results in membrane rupture, cell swelling, and the activation of inflammasomes, particularly NLRP3 [11]. In the second mechanism, the formation of antigen–antibody complexes is associated with elevated levels of interleukin 8 (IL-8) and chemoattractant proteins [8]. The first mechanism involves multiple pathways, one of which is the activation of the NLRP3 inflammasome. Once the NLRP3 sensor protein detects viral RNA or proteins via DAMPs or PAMPs, it oligomerises and activates its effector, caspase-1. Caspase-1, a cysteine protease enzyme, activates the pro-inflammatory cytokines IL-1β and IL-18 and cleaves Gasdermin D. IL-1β and IL-18 subsequently activate the NF-κB and JNK signalling pathways. NF-κB increases the transcription of pro-inflammatory genes, such as interferon-gamma (IFN-γ), while JNK triggers the cellular stress response. Together, these pathways amplify inflammatory responses, contributing to the cytokine storm [12]. This inflammatory form of cell death releases intracellular contents, including pro-inflammatory cytokines, which contribute to the cytokine storm [12]. IFN-γ is a critical antiviral cytokine produced by many cell types, with plasmacytoid dendritic cells (pDCs) being a major source. IFN-γ activates both antiviral and pro-inflammatory pathways, including the induction of interferon-stimulated genes (ISGs). ISGs operate through two distinct pathways depending on the concentration of IFN-γ. At low levels of IFN-γ, ISGs encode proteins such as protein kinase R, which inhibits viral protein synthesis and replication. However, at high levels of IFN-γ, as observed in patients with COVID-19, IFN-γ drives the secretion of pro-inflammatory cytokines and chemokines, further amplifying the inflammatory response [13]. Thus, the timing and localisation of interferon (IFN) responses are crucial in shaping their role in COVID-19. An early, localised IFN response in the respiratory tract can help control SARS-CoV-2 infection and prevent severe disease. In contrast, delayed or systemic IFN responses may result in excessive inflammation, contributing to disease progression and viral dissemination [13]. As mentioned above, the second mechanism involves adaptive immunity. In this process, the body produces neutralising antibodies targeting the virus’s surface antigens. Immunoglobulin (Ig) G antibodies bind to the viral S protein and subsequently engage Fc receptors on monocytes and macrophages. This interaction leads to the recruitment of pro-inflammatory monocytes and macrophages to the lungs through the action of IL-8 and monocyte chemoattractant protein-1 (MCP-1) [8]. This hyper-inflammatory response can lead to severe symptoms in patients with COVID-19, including heart failure, acute kidney injury, and damage to the cardiac and respiratory systems, as a result of widespread cytokine attacks on the respiratory, renal, coagulation, and cardiovascular systems [14]. This review examines the impact of SARS-CoV-2 on red blood cell (RBC) production, alterations in RBC morphology, and the broader haematopoietic system [6,8].

It highlights the role of key regulatory factors such as hepcidin, erythroferrone (ERFE), and growth differentiation factor 15 (GDF-15) in anaemia and erythropoiesis. Additionally, this review explores how SARS-CoV-2 triggers cytokine storms through interleukins, immature blood cells, and CD71+ erythroid cells, providing novel insights into the management of haematological manifestations and immune responses in patients with COVID-19.

## 2. Hepcidin and Inflammation

**Figure 1:**
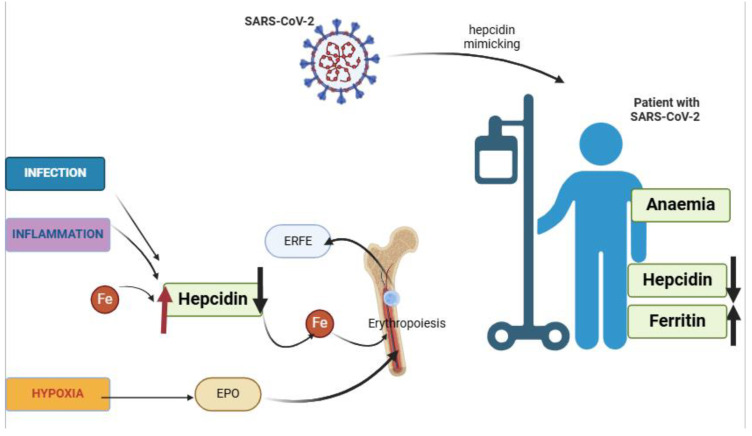
Opposing effects of hypoxia and inflammation on hepcidin synthesis. EPO—erythropoietin; ERFE—erythroferrone; Fe—iron; SARS-CoV-2—severe acute respiratory syndrome coronavirus 2.

Inflammation and hypoxia interact in complex ways [15]. The oxygen deficiency seen in patients with COVID-19 disrupts erythropoiesis and induces high levels of inflammation [16]. Due to its effects on the lungs, COVID-19 leads to reduced oxygen levels in the blood, resulting in hypoxia [17]. Both hypoxia and inflammation influence hepcidin regulation, with hypoxia generally exerting a stronger effect (Figure 1) [18]. Specifically, hypoxia inhibits hepcidin, while inflammation increases its production [19]. Hepcidin, an immune system protein, decreases circulating iron levels by interacting with ferroportin, a transporter on the cell membrane that inhibits iron release from cellular storage [20]. This is crucial during infection, as both pathogens and the host utilise iron [21].

Hepcidin synthesis is enhanced by high iron levels, infection, and inflammation, but inhibited by erythropoiesis in the bone marrow via ERFE [18,22] and growth differentiation factor-15 (GDF-15) [23]. During infection, ERFE levels are typically very low. ERFE, a hormone produced by erythroblasts in the bone marrow [24], plays a crucial role in regulating iron metabolism, particularly during increased RBC production, such as in anaemia or after blood loss [25]. ERFE inhibits hepcidin production, increasing iron availability for erythropoiesis [26]. Hypoxia also stimulates erythropoietin (EPO) production, which enhances erythropoiesis and ERFE synthesis [27]. This regulatory cascade ensures iron availability, haemoglobin (Hb) production, and the formation of new RBCs [21].

Studies have shown that during infection, hepcidin anti-microbial peptide (HAMP) mRNA and hepcidin protein levels are elevated [18,28]. However, hypoxia increases ERFE production, which should inhibit hepcidin [29]. In patients with COVID-19, a positive correlation between ERFE and hepcidin levels has been observed, generating a paradox. During inflammation, iron recycling from senescent RBCs [18,28] is impaired due to the increased binding of hepcidin to ferroportin, causing hypoferremia and anaemia [18,30]. High hepcidin levels keep iron trapped in stores, resulting in reduced circulating iron and a condition of quasi-functional iron deficiency. Upon hospital admission, most patients with COVID-19 were anaemic, with an average Hb concentration of 11.6 g/dL [18]. Maira et al. reported that ICU patients were more likely to develop moderate anaemia than non-ICU patients. At the initial Hb measurement (time zero, T0), non-ICU patients had higher Hb levels compared to ICU patients [18]. Although Hb levels in non-ICU patients decreased over time, they remained within the lower normal range (mean of 12.8 g/dL at T0 and 12.4 g/dL at a subsequent point or T1). The ICU group experienced a more severe decline in Hb level (mean of 11.6 g/dL at T0 and 10.3 g/dL at T1) [18], with 46.7% of ICU patients being anaemic compared to 35.7% of non-ICU patients [18,31,32]. Despite severe inflammation and high levels of inflammatory markers and interleukins, ICU patients exhibited surprisingly low circulating hepcidin levels, accompanied by high ferritin levels [18,22].

In a similar study by Gugo et al. [21], normoxic patients with COVID-19 showed increased levels of hepcidin, ferritin, and EPO. Chakurkar et al. found that baseline hepcidin and ferritin levels were positively associated with negative outcomes, such as mortality, mechanical ventilation, and kidney damage [33]. The authors hypothesised that the SARS-CoV-2 virus might mimic hepcidin, suppressing its expression in the liver. This is supported by sequence similarities identified in structural models between the hepcidin molecule and the cytoplasmic tail of the SARS-CoV-2 spike glycoprotein [21].

## 3. Erythropoiesis Level in COVID-19

A high level of immature blood cells, commonly seen in haemolytic anaemia, iron-deficiency anaemia, and respiratory failure, was present in the blood of patients with COVID-19 [18,34,35]. Upon admission, lower EPO levels were observed, likely due to the inhibitory effect of inflammatory cytokines, including IL-6 and tumour necrosis factor-alpha (TNF-α), on both EPO production and erythroid progenitor cells. The overproduction of cytokines and the dysregulation of iron metabolism suppress EPO by downregulating its production in the kidneys, leading to erythroid progenitor cell apoptosis, a reduction in EPO receptors, and the inhibition of cell growth [36]. Taneria et al. noted that a viral invasion of the bone marrow further inhibits erythropoiesis [37]. Several mechanisms contribute to the downregulation of erythropoiesis. The production of IL-6 and TNF-α negatively impact erythropoiesis by inhibiting kidney-produced EPO and stimulating hepcidin, which regulates iron homeostasis [38]. Additionally, TNF-α, IL-1B, and IFN-γ inhibit the proliferation of erythroid progenitor cells and disrupt the bone marrow’s erythropoiesis-supporting environment. Another mechanism involves oxidative stress, inflammatory mediators, and apoptosis induction, through the Fas ligand (FasL), reactive oxygen species (ROS), and nitric oxide (NO), which trigger the apoptosis of erythroid progenitor cells [38].

It was also observed that the more severe the anaemia, the faster the EPO concentration increased in the blood, doubling in just a few days [18,21]. This suggests that the initial hypoxic stimulus was stronger and more sustained than the inflammatory response [18,28]. Gugo et al. noted no reduction in hepcidin levels, consistent with Pasricha et al.’s findings that erythropoiesis itself, rather than EPO alone, is the key factor in suppressing hepcidin expression [21,37].

## 4. Wuhan Strain Effects on Circulating CD71+ Erythroid Cells

Among the various SARS-CoV-2 strains (Wuhan, Delta, and Omicron), the Wuhan strain had the greatest impact on haematopoiesis [28]. A study by Saito et al. examined the effects of different SARS-CoV-2 variants on erythropoiesis. They found that patients infected with the original Wuhan strain had a higher frequency of circulating CD71+ erythroid cells (CECs) compared to those infected with the Delta and Omicron variants. This suggests that the Wuhan strain may have a more pronounced impact on haematopoiesis. The virus disrupts haeme metabolism, affects Hb function and oxygen transport, and causes morphological changes in RBCs, reducing their oxygen-carrying capacity [28,35,36,39]. Studies have also documented that different SARS-CoV-2 strains affect erythropoiesis and CECs, including specific markers like CD71+ and CD45+ [28,34,35]. CD71, a marker for the transferrin receptor, indicates metabolically active or progenitor endothelial cells, while CD45 is a leukocyte marker [28,34,35]. The main target of the different COVID-19 strains is the angiotensin-converting enzyme 2 (ACE2) receptor and its co-receptor, transmembrane protease, serine 2 (TMPRSS2). ACE2, a protein expressed in various cell types (lungs, heart, kidney), regulates the renin–angiotensin system. The viral spike protein binds to ACE2 to initiate cell entry, while TMPRSS2, a serine protease enzyme found on the surface of host cells, cleaves and activates the viral spike protein after it binds to ACE2 [34,35,36]. The expression of ACE2 and TMPRSS2 on CECs suggests that SARS-CoV-2 targets these cells and invades erythroid progenitors. This process disrupts ACE2’s protective role [28,34,35,36], contributing to hypoxia [18]. Additionally, high cytokine levels induce haematopoietic stress, increasing CEC release into the bloodstream. The large number of immature RBCs and CECs released into circulation become targets for SARS-CoV-2, which leads to their depletion via lysis or phagocytosis. This phenomenon likely contributes to the anaemia observed in all patients with COVID-19 [28,34,35,36]. A possible therapeutic approach involves dexamethasone, which reduces ACE2/TMPRSS2 expression in CECs. Studies suggest that immunosuppressive drugs like dexamethasone may modulate CEC activity, dampen the overstimulation of the immune response, and offer protection against viral infection [28,34,35,36]. This highlights the importance of timing and efficacy when using such interventions. Moreover, the impact of SARS-CoV-2 on platelets has revealed that thrombocytopenia is a common feature in patients with COVID-19. Elevated markers of platelet activation point to a crucial role for platelets in the body’s response to the virus, contributing to the thrombotic complications often seen in severe COVID-19 cases. Investigating the relationship between the virus and haematopoiesis could inform the development of targeted therapeutic strategies for managing various COVID-19 symptoms [28,34,35,36,40].

## 5. Circulating CD71+ Erythroid Cell Depletion

Studies investigating COVID-19’s impact on haematopoiesis have identified a significant increase in CD71+ erythroid cells in the peripheral blood of patients, particularly those with moderate to severe disease [34,35]. This expansion correlates with disease severity and a higher likelihood of ICU admission, suggesting that CECs could serve as potential indicators of COVID-19 progression. CD71+ and CD45+ cells exhibit immunosuppressive effects, which negatively impact CD8+ T-cell functions, thereby weakening the adaptive immune response against the virus [28,34,35,36]. Furthermore, the immunomodulatory properties of CECs, including their ability to suppress T- and B-cell functions, have been highlighted. Notably, CECs from patients with COVID-19 show an increased expression of arginase I/II and ROS, contributing to global immunosuppression and impairing key immune cell functions in vitro [28,40]. These findings underscore the dual role of CECs, initially acting as protective agents against hyperinflammation but later compromising T-cell functions and antibody production as the disease progresses [41].

## 6. SARS-CoV-2, Cytokine Storm, and Erythropoiesis

**Figure 2:**
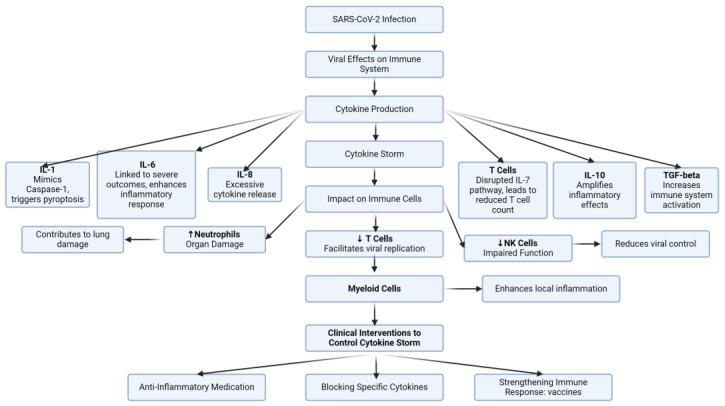
SARS-CoV-2 and cytokine storm.

Recent research has revealed multiple effects of SARS-CoV-2 on haematopoiesis, impacting blood cell production and function. For example, while SARS-CoV-2 increases neutrophils, natural killer (NK) cells, IL-6, and IL-8, it decreases T-cell levels [42,43]. The correlation between SARS-CoV-2 and IL-6 levels requires further clarification, as it reflects different stages or contexts of infection. Early in infection, SARS-CoV-2 may transiently suppress the production of IL-6 and other pro-inflammatory cytokines in specific cell types, such as T cells, as part of its immune evasion strategy [44]. However, in severe COVID-19 cases, dysregulated immune activation leads to a hyper-inflammatory state known as a “cytokine storm” [45,46]. This state results in the excessive production of IL-6, among other cytokines, by immune cells such as macrophages and monocytes [47]. Elevated IL-6 levels in the blood are a hallmark of this hyper-inflammatory phase and are strongly associated with poor outcomes, including organ damage and increased mortality. Thus, while SARS-CoV-2 may initially reduce IL-6 levels, the later uncontrolled inflammatory response drives significant IL-6 overproduction, contributing to disease severity. Elevated levels of ferritin, IL-6, IL-7, and IL-10 in the blood are major indicators of higher mortality risk. Interleukin 1 (IL-1) plays a key role in the inflammatory response to infection. Normally, the enzyme caspase-1 converts inactive IL-1 into its active form. However, it has been observed that SARS-CoV-2 can increase IL-1 levels by mimicking caspase-1 activity, inducing pyroptosis and triggering a cytokine storm [42]. This increase is associated with symptoms related to patients with COVID-19, such as intravascular coagulation and thrombotic events. The virus impacts IL-7 in two primary ways: by impairing the IL-7 signalling pathway and by reducing T-cell numbers. IL-7 is a protective cytokine that, upon binding to its receptor (IL-7R), activates the JAK/STAT signalling pathway. This pathway prevents T-cell apoptosis by upregulating anti-apoptotic proteins. A reduced IL-7 level is generally associated with decreased T-cell survival and lower T-cell counts [30,31,32]. In severe cases, particularly in ICU patients with COVID-19 (as observed by Huang et al. [14]), the excessive production of IL-7 leads to the downregulation of its receptor on T cells. This receptor downregulation reduces IL-7 consumption by T cells, weakening its anti-apoptotic effects. This imbalance results in immune dysregulation and a lymphopenic state, wherein the body produces more IL-7 in a compensatory response. This feedback loop further exacerbates disease severity. Given this knowledge, IL-7 can serve as a biomarker for COVID-19 severity and a potential therapeutic tool. Studies in cancer and HIV have demonstrated that recombinant human IL-7 (rhIL-7) can promote T-cell proliferation and survival by activating anti-apoptotic pathways. Additionally, IL-7 enhances the function of T follicular helper (Tfh) cells, which are critical for B-cell activation and antibody production in response to vaccines. This insight has opened up new therapeutic possibilities for respiratory virus vaccines. Combining IL-7 with vaccines may enhance vaccine efficacy for SARS-CoV-2 by boosting immune responses and improving T-cell survival [48]. Such strategies offer a promising avenue for increasing protection against COVID-19 and other respiratory viruses [49]. Another key interleukin, IL-10, is crucial in determining disease severity [50]. It is an anti-inflammatory interleukin that can inhibit the immune response, but under some circumstances, it can exacerbate inflammation by regulating macrophage genes and functions by activating Signal Transducer and Activator of Transcription 3 (STAT3), which drives the production of suppressors of cytokine signalling proteins, SOCS1 and SOCS3 [51]. In the case of hypoxic conditions usually seen in patients with COVID-19, the effect of IL-10 on macrophages can be altered and cause an increase in pro-inflammatory cytokines like IL-6 via the activation of the hypoxia-inducible factor (HIF) pathway [48]. HIF can regulate genes and pathways, for example, by upregulating TNF-α production in macrophages, especially in the presence of inflammatory stimuli, leading to a feedback loop of inflammation [52]. It can also, in severe COVID-19 cases, increase ROS production, causing oxidative stress and tissue damage such as in lung tissues. IL-10 is an important factor as it has a dual effect. In both cases, the IL-10 overproduction seen in patients with COVID-19 causes more damage than good. Excessive levels of IL-10 could, on the one hand, suppress immune function, leading to viral persistence, and could, on the other hand, exacerbate inflammation, causing more damage. IL-17 also contributes to the inflammatory response through T helper 17 cells. Antigen-presenting cells present the viral component to Th17 cells, leading to IL-17 secretion. It then attracts immune cells (monocytes and neutrophils) to infection sites, such as the lungs, and causes inflammation. IL-17 is also implicated in the activation of other cytokines (IL-6, IL-8, and TNF-α), which intensifies inflammation. This correlates with the symptoms seen in patients with COVID-19, such as acute respiratory distress syndrome (ARDS) and multi-organ damage. TNF-α plays a critical role in COVID-19’s inflammatory response, especially in respiratory issues and lung damage. It causes bronchial hyperresponsiveness, narrowing airways and increasing neutrophil activity in the respiratory epithelium, promoting cytokine production and causing lasting lung damage via pulmonary fibrosis. It can also induce MMP-9 release by neutrophils, further damaging lung tissue. In COVID-19, the level of TNF-α in the blood is elevated, as monocytes, macrophages, B cells, T cells, and fibroblasts produce it to intensify inflammation. TNF-a inflammatory effects are activated via two receptors: TNFR1 and TNFR2. Once it binds its receptors, TNF-α is able to activate different pathways such as NF-kB, MAPK, and apoptosis. Each of these pathways increases inflammation and the immune response in different ways. For example, NF-kB can increase pro-inflammatory genes, and MAPK promotes cell survival, proliferation, and differentiation [53]. SARS-CoV-2 also causes behavioural changes in neutrophils, which can lead to collateral damage in vital organs, especially in the lungs, a key indicator of disease severity [22,54,55]. In addition, SARS-CoV-2 increases the level of transforming growth factor β (TGF-β), which is involved in tissue homeostasis, immune regulation, and wound healing. Elevated TGF-β activity in response to SARS-CoV-2 enhances immune activity, potentially preventing autoimmune diseases and chronic inflammation [54]. Beyond neutrophils and T cells, SARS-CoV-2 also affects NK cells, impairing their ability to inhibit viral replication [56]. Consequently, this dysregulation allows the virus to replicate more efficiently and increases cytokine production [49,54,57,58]. Severe cases have also revealed distinct changes in myeloid cells, such as macrophages and monocytes, which amplify local inflammation and may contribute to disease progression [42]. The combined action of these mechanisms culminates in the cytokine storm (Figure 2). Various clinical interventions, including antagonists, antibodies, and medications such as Tocilizumab, IL-7 as a vaccine adjuvant, and other cytokine inhibitors, have been shown to help control this storm [49,54,57,58]. The cytokine storm also disrupts iron metabolism and erythropoiesis via its effect on hepcidin regulation. High levels of interleukins (IL-6, IL-1β, IL-10), TNF-α, and IFN-γ trigger increased hepcidin production and impair erythropoietin. Some interleukins, such as IL6, can increase hepcidin production in the liver. The increase in hepcidin therefore decreases iron availability. This limitation of iron negatively affects RBC production, leading to anaemia and hypoferremia. To conclude, the summation of cytokine storm effects, CEC depletion, and the increase in hepcidin level negatively affect erythropoiesis and increase COVID-19 effects.

## 7. GDF-15 and ERFE’s Effect on Hepcidin

Both GDF-15 and ERFE suppress hepcidin expression, impacting iron homeostasis. GDF-15 belongs to the TGF-β superfamily, while ERFE, involved in erythropoiesis, is not part of the tumour necrosis factor-α (TNF-α) family. ERFE regulates hepcidin expression during increased erythropoiesis by inhibiting it to ensure sufficient iron availability for RBC production. Both GDF-15 and ERFE can suppress hepcidin expression via the bone morphogenetic protein (BMP)–Smad pathway, which plays a role in cell growth, tissue development, and differentiation. By inhibiting hepcidin, these regulators influence iron metabolism during various disease states. Few studies have explored the link between GDF-15, ERFE, and viral infections. For instance, research has demonstrated an inverse correlation between hepcidin and ERFE/GDF-15 under different conditions. Delaye et al. observed the expected inverse correlation: lower ERFE and GDF-15 levels were associated with higher hepcidin concentrations, consistent with the established mechanism by which GDF-15 and ERFE suppress hepcidin expression [59]. This finding aligns with the general understanding that hepcidin and ERFE/GDF-15 usually exhibit an inverse relationship, as GDF-15/ERFE typically inhibits hepcidin release and activity [59]. However, in this study, patients with COVID-19 displayed a non-paradoxical disruption of this usual inverse relationship. Hepcidin levels were significantly higher in patients with COVID-19 compared to those with non-COVID-19 inflammatory diseases. This elevated hepcidin level correlated with low serum iron levels in patients with COVID-19, a pattern also observed in other inflammatory diseases, particularly pulmonary diseases. This is clinically significant, as low serum iron levels in the blood are associated with higher mortality rates and account for many of the symptoms seen in patients with COVID-19. Zhao et al. noted that this phenomenon could be explained by the pathophysiology of hepcidin, which leads to increased intracellular iron concentrations [60]. This increase triggers ferroptosis, a form of regulated cell death associated with lipid peroxidation, which, in turn, induces an immune response and cell death [59]. Furthermore, this condition of increased intracellular iron, particularly within macrophages, can lead to the so-called ‘macrophage activation syndrome’, characterised by the massive release of ferritin-bound iron into circulation [61]. This release, often triggered by inflammation, transfusions, or infection, activates macrophages and results in a highly toxic environment for cells and organs [62]. The preferential accumulation of iron in macrophages has also been consistently described in bone marrow biopsies of patients with COVID-19 [4].

## 8. Morphological Changes and Effects

In addition to the points discussed above, studies have observed morphological changes in the RBCs of patients with COVID-19 that impair oxygen delivery [34,39]. A high number of spiculated cells, resembling echinocytes and acanthocytes, were detected, indicating membrane alterations primarily caused by changes in lipid and protein composition [34,39]. Ferritin’s heavy chain (FTH1) was also affected by the oxidative stress associated with the disease, impairing its role in maintaining cytoskeleton integrity and its ability to modulate iron storage and release [8,18]. Various surface markers and functional alterations, such as viral antigens on RBC surfaces and complement activation products, were detected in patients [34,39]. Lipidomic, proteomic, and metabolomic analyses have revealed structural membrane abnormalities and alterations in lipid metabolic pathways in RBCs [39,63]. The endothelial damage caused by SARS-CoV-2 infection, coupled with oxygen uptake dysfunction, leads to a rise in hypoxia and anaemia [64]. One proposed mechanism for the decrease in Hb oxygen affinity is the marked increase in 2,3-bisphosphoglycerate (2,3-BPG), a glycolysis by-product that shifts the oxygen dissociation curve to the right [64,65]. As an allosteric effector, 2,3-BPG binds to Hb’s beta subunits, reducing its oxygen affinity and promoting oxygen release. Consequently, while this mechanism facilitates oxygen unloading in tissues, the overall oxygen-carrying capacity of RBCs may be impaired, contributing to COVID-19-induced anaemia and ARDS. The primary cause of the low oxygen-carrying capacity, however, is the reduced circulating haemoglobin concentration. Notably, some studies suggest that COVID-19 does not significantly affect Hb’s oxygen affinity [66,67], highlighting the need for further investigation. Patient RBC protein analyses could help confirm changes in oxygen affinity, and studies on drugs that enhance Hb’s oxygen affinity may provide promising strategies to mitigate the haematological effects of COVID-19.

## 9. Clinical Haematological Findings Stemming from SARS-CoV-2 Infection

Coronaviruses have historically been associated with blood cell abnormalities. SARS-CoV-1 and Middle East respiratory syndrome coronavirus (MERS-CoV) are precursors to SARS-CoV-2. SARS-CoV-1 is notably linked with a high incidence of thrombocytopenia (20–55%), a marked decrease in platelet count, and lymphopenia (69.6–100%), a deficiency in white blood cells [68]. In comparison, SARS-CoV-2 causes thrombocytopenia in 5–21% and lymphopenia in 30–75% of patients with COVID-19 [69]. Coagulopathic markers, such as D-dimer levels, also indicate abnormal blood clot breakdown in 45% of patients with SARS-CoV-1 [70]. Patients with MERS-CoV similarly exhibited thrombocytopenia (31–40%) and lymphopenia (44–60%) [71]. In SARS-CoV-2 infection, elevated D-dimer levels (≥2  µg/mL) were found in 48% of patients, who also required an additional 31 L/min of oxygen [72]. Patients with D-dimer levels below 2 µg/mL had a 76% lower risk of death during hospitalisation [72]. Unlike coronaviruses that cause the common cold, SARS-CoV-1, MERS-CoV, and SARS-CoV-2, which cause severe respiratory complications, clearly manifest haematological abnormalities [73]. In SARS-CoV-2 infection, these haematological changes serve as indicators of disease severity, hospitalisation risk, and mortality [74]. Coagulation dysfunction is common in COVID-19, primarily due to the antiviral inflammatory response, the viral invasion of pericytes, and the increased presence of neutrophils in the alveoli [69,75]. Vascular endothelial inflammation, triggered by the dysregulation of ACE2 receptors and angiotensin II accumulation, plays a key role in these haematological effects [76]. ACE2 receptors are expressed across various organs’ endothelial cells, leading to the widespread migration of immune cells and cytokine release at the endothelial level [77,78]. This dysregulated endothelium and procoagulant state results in blood vessel damage, thrombocytopenia, neutropenia, and excessive clotting (thrombi formation and platelet consumption) [69,77,79]. These effects persist in post-acute long-term COVID-19 syndrome [77]. Moreover, the disease increases the secretion of the procoagulant von Willebrand factor (VWF) due to endothelial cell damage [80]. These haematological changes lead to hypoxia, clotting, and thrombi formation in both arteries and veins, causing conditions such as pulmonary embolism, deep vein thrombosis, and ischaemic strokes, all linked to elevated D-dimer levels [74]. D-dimer fragments, formed from dissolving clots, serve as a key predictor of disease progression, coagulopathies, and survival. Deceased patients with COVID-19 had a mean D-dimer level of 5.46 μg/mL compared to 1.55 μg/mL in recovered and discharged patients [74,76,79]. Extensive research has shown that SARS-CoV-2 has long-term effects on multiple systems, particularly the haematological system. A 12-month longitudinal study on individuals who tested positive for SARS-CoV-2 revealed an increased incidence of thrombotic events as part of the downstream cardiovascular impact of COVID-19. In a cohort of male patients from Spanish electronic medical records, the incidence of arrhythmias, thrombosis, and heart failure was elevated, with hazard ratios between 1.26 and 1.35. The study included conditions such as atrial fibrillation, flutter, and tachycardia. Overall cardiovascular mortality was also higher in individuals who had tested positive for SARS-CoV-2 during the follow-up period. Researchers attribute this increased cardiovascular risk to persistent microvascular thrombosis, inflammation, and endothelial dysfunction from the acute phase of infection [45].

## 10. Avenue for Treatment: Targeting iRhom2 for Cytokine Storm Management

The leading cause of mortality in individuals with COVID-19 is acute respiratory distress syndrome (ARDS), which is driven by a cytokine storm involving IFN-γ, granulocyte colony-stimulating factor (G-CSF), and TNF-α. TNF-α, in particular, plays a major role in the inflammation associated with ARDS by promoting the release of additional pro-inflammatory cytokines [81]. Pereira et al. explored the potential to mitigate the cytokine storm through the inhibition of ADAM17 (A Disintegrin and Metalloproteinase 17), a key metalloprotease that cleaves IL-6R, IL-8, ACE-2, and TNF-α, all contributors to the post-acute sequelae of SARS-CoV-2 infection. The study focused on blocking ADAM17 using iRhom2 (inactive Rho GTPase homolog 2), a crucial rhomboid protease required for ADAM17 maturation specifically in immune cells. When human bronchial epithelial cells infected with SARS-CoV-2 were transfected with iRhom2 inhibitors, there was a significant reduction in the release of cytokines (IL-6, IL-8, IFN, and TNF-α) involved in the cytokine storm. This suggests that iRhom2 inhibition could be a promising approach for treating cytokine storms, potentially reducing the respiratory and haematological complications associated with long-term COVID-19 [82].

## 11. Conclusions and Further Perspectives

**Figure 3:**
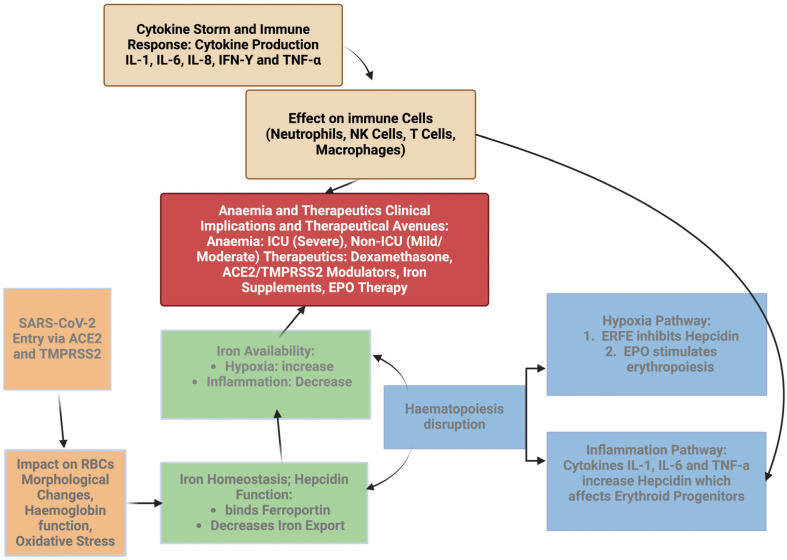
This figure illustrates the multifaceted impact of COVID-19 on haematopoiesis and red blood cell health. SARS-CoV-2 triggers inflammation and hypoxia, leading to elevated levels of hepcidin. Increased hepcidin suppresses iron absorption and release, impairing erythropoiesis and reducing red blood cell production. Simultaneously, diminished levels of erythroferrone (ERFE) fail to counterbalance hepcidin’s effects, exacerbating iron restriction. The combined effects of disrupted iron homeostasis, red blood cell dysfunction, and the cytokine storm result in anaemia, further complicated by impaired oxygen transport and systemic inflammation.

The identified connections between viral infection, haematological changes, and disease severity open new avenues for therapeutic interventions targeting the haematopoietic system (Figure 3) [83].

Exploring novel treatment strategies and mitigating complications arising from COVID-19 haematological effects is a crucial step forward [84]. The insights gained from studying COVID-19’s haematological manifestations not only enhance our understanding of the disease’s pathophysiology but also guide future research (Table 1). One critical area is the need for in-depth investigations into the long-term effects of COVID-19 on haematopoiesis and its potential latent impacts on individuals recovering from the disease. Longitudinal studies could provide valuable insights into persistent haematological changes and their implications for overall health. In addition, the research community should prioritise developing targeted therapies specifically addressing COVID-19’s haematological aspects, aiming to improve patient outcomes and alleviate long-term consequences. Further research should also explore the systemic effects of COVID-19, given the intricate connections between the haematopoietic system and other organs. A holistic approach could reveal additional complexities in the disease’s pathogenesis and offer a more comprehensive understanding of how COVID-19 affects various physiological processes. Such knowledge is essential for refining clinical management strategies, improving treatment protocols, and preparing healthcare systems to manage the multifaceted impact of COVID-19 on the haematopoietic system. This review highlights the complex interplay between hepcidin, inflammation, and haematopoiesis in COVID-19 pathophysiology. SARS-CoV-2 has been shown to dramatically affect the haematopoietic system, altering erythropoiesis and iron metabolism. The regulation of hepcidin by inflammation and hypoxia significantly influences RBC production and function. Elevated hepcidin levels, driven by inflammation and hypoxia, contribute to the anaemia seen in severe cases, with diminished ERFE levels exacerbating this effect. The disease’s impact on RBC morphology, oxygen transport, and overall haematological health underscores the multifaceted challenges posed by COVID-19. CECs and immature blood cells are critical in understanding disease severity and progression. Different SARS-CoV-2 strains, such as the Wuhan strain, have varying impacts on haematopoiesis and RBC function, contributing to anaemia. Finally, the cytokine storm triggered by SARS-CoV-2 further complicates the immune response and exacerbates both local and systemic inflammation.

## 12. Methods

A literature search was conducted using PubMed, Web of Science, SCOPUS, Embase, and Google Scholar to identify studies on haematological findings in COVID-19 up to December 2024. The following keywords were used: SARS-CoV-2, COVID-19, clinical findings, laboratory findings, white blood cells, hepcidin, erythropoiesis, haematology, coagulation, iron metabolism, hypoxia, and RBC count. The initial selection was based on article titles and abstracts, followed by a full-text review. The references from the full-text articles were examined to obtain further relevant studies. After the removal of duplicates, articles pertinent to the topic were included. The findings from primary research articles, case reports, and case series are summarised and discussed. The inclusion criteria were as follows: publications in peer-reviewed journals, articles in English, and a focus on COVID-19/SARS-CoV-2 pathophysiology, hepcidin, iron metabolism, cytokine responses/storm, white blood cells, erythropoiesis, haematology, coagulation, RBC count, clinical findings, or laboratory findings. The exclusion criteria included preprints, opinion pieces without publication dates, duplicate publications, and conference abstracts.

## Figures and Tables

**Table 1:** Table summarising the effects found in different experimental studies.

Participants	Methods	Key Findings	Therapeutic Findings	Study
Mouse models	Gene expression analysis, protein analysis, tissue culture	HIF regulates hepcidin through EPO-stimulated erythropoiesis	Identified HIF pathway as potential therapeutic target for iron disorders; no COVID-specific therapeutic findings	Laboratory research [19]
Human monocytes/macrophages	Cell culture, signalling pathway analysis	IL-4 and IL-13 use distinct signalling pathways in macrophage activation	Identified potential therapeutic targets in macrophage activation pathways	Laboratory research [85]
41 hospitalised patients with confirmed COVID-19	Clinical data collection, laboratory tests, chest CT scans	98% had a fever, 76% had a cough, 44% had fatigue; lymphopenia in 63%; all had pneumonia	Oxygen therapy (100%), mechanical ventilation (10%), ECMO (2%); antiviral treatment showed no significant benefits	Clinical study [14]
Patients with COVID-19 (number not specified in citation)	Large-scale plasma proteomics analysis	Identified new molecules and mechanisms in host response to SARS-CoV-2	Multiple new therapeutic targets identified through proteomics	Clinical proteomic study [63]
259 patients with COVID-19	Blood tests, iron parameter analysis	24.7% of patients had anaemia; disturbed iron homeostasis common in severe cases	Iron parameters could serve as predictive markers; suggested potential benefit of iron supplementation	Prospective clinical study [31]
50 patients with COVID-19	Iron status measurements, clinical outcome tracking	Hypoferremia associated with increased hospitalisation and oxygen needs	Suggested iron status as a therapeutic target; no direct intervention tested	Clinical observational study [32]
139 patients with COVID-19 + controls	Multiomics analysis (transcriptomics, proteomics), clinical data	Identified megakaryocyte and erythroid cell responses as disease hallmarks	Revealed potential therapeutic targets in cellular response pathways	Longitudinal multiomics study [40]
Patients with COVID-19 (number not specified in citation)	Blood gas analysis, oxyhaemoglobin dissociation curve measurements	Left shift in oxyhaemoglobin dissociation curve in severe COVID-19	Implications for oxygen therapy management	Clinical study [65]
40 patients with COVID-19	T-cell analysis, functional immune studies	Elevated T-cell exhaustion and reduced functional diversity in severe cases	Suggested immunotherapy approaches; no direct therapeutic testing	Clinical study [41]
Patients with severe COVID-19	T-cell homeostasis and function analysis	Profound dysregulation of T-cell homeostasis and function	Suggested immunomodulatory therapeutic approaches	Clinical immunology study [58]
4 patients with COVID-19	Clinical observation, blood tests, oxygen saturation monitoring	Successfully treated severe COVID-19 hypoxia with erythropoietin	EPO treatment improved oxygen saturation and clinical outcomes; suggested as potential therapy for severe COVID-19 hypoxia	Case series + literature review [16]
Human erythroid progenitor cells	Cell culture, viral infection analysis, molecular studies	Demonstrated direct infection of erythroid progenitors by SARS-CoV-2	Suggested protective strategies for erythroid progenitors as therapeutic approach	Laboratory research [35]
134 hospitalised patients with COVID-19	Blood tests, hepcidin measurements, clinical outcome tracking	High hepcidin levels predicted disease severity and mortality	Suggested hepcidin as potential therapeutic target and prognostic marker; no direct therapeutic interventions tested	Clinical cohort study [30]
Samples of human erythroid cells and patients with COVID-19	Cell culture, viral infection studies, immune response analysis	SARS-CoV-2 can infect erythroid cells; these cells suppress adaptive immunity	Identified potential therapeutic target in protecting erythroid cells; immunomodulation possibilities	Laboratory research [34]
Patients with COVID-19	Blood analysis for erythroid regulators and hepcidin	Specific changes in erythroid regulators and hepcidin levels	Suggested targeting erythroid regulation pathways	Clinical study [59]
123 patients with COVID-19	Blood sampling, iron metabolism markers, inflammatory markers	Iron metabolism alterations correlated with disease severity; abnormal hepcidin levels	Suggested potential therapeutic targeting of iron metabolism pathways; no direct therapeutic intervention tested	Prospective clinical study (IRONCOVID) [18]
27 hospitalised patients with COVID-19	RBC morphology analysis, blood smear examination	Significant RBC morphologic abnormalities in patients with COVID-19	Morphological changes could guide therapeutic strategies; no direct intervention tested	Clinical study [39]
Cell cultures	In vitro infection with different SARS-CoV-2 variants, gene expression analysis	Different variants had varying effects on erythropoiesis pathways	Identified variant-specific effects on erythropoiesis; suggested potential need for therapeutic approaches	Laboratory research [28]
Non-anaemic patients with COVID-19 (number not specified in citation)	Blood tests, inflammatory markers, hepcidin measurements	Correlation between hypoxia severity and hepcidin levels	Suggested hepcidin as therapeutic target; no direct therapeutic interventions tested	Clinical study [21]
Patients with COVID-19	Mixed-effect modelling to analyse oxyhaemoglobin dissociation curve	Investigated changes in oxyhaemoglobin dissociation curve in patients with COVID-19	No specific therapeutic recommendations	Observational cohort [66]
Patients with COVID-19	Analysis of haemoglobin O_2_ affinity	No relevant clinical effects of SARS-CoV-2 on haemoglobin O_2_ affinity	Standard oxygen therapy remains appropriate; no need for specialised oxygen delivery modification	Clinical study [67]
Patients with SARS in Hong Kong	Analysis of major SARS outbreak	Documented major outbreak characteristics and clinical manifestations	Early identification and isolation crucial for treatment success	Clinical outbreak study [70]
Patients with MERS	Analysis of haematologic, hepatic, and renal function	Documented changes in multiple organ systems in patients with MERS	Suggests multi-organ monitoring approach for treatment	Clinical study [86]
Patients with COVID-19	Analysis of D-dimer trends	D-dimer trends can predict prognosis for patients with COVID-19	D-dimer monitoring can guide therapeutic decisions and intensity of care	Retrospective chart review [72]
COVID-19 survivors and non-survivors	Biochemical analysis of ferritin and D-dimer	Compared ferritin and D-dimer levels between survivors and non-survivors	Suggests using ferritin and D-dimer levels to guide treatment intensity	Clinical analysis [74]
Patients with severe COVID-19	Clinical observation	Severe COVID-19 associated with endothelial activation	Suggests potential benefit of endothelial-targeted therapies	Case report [76]
N/A	Comprehensive review of thrombotic complications	Reviewed implications for prevention and antithrombotic therapy	Detailed guidelines for anticoagulation in patients with COVID-19	State-of-the-art review [77]
Hospitalised patients with COVID-19	Analysis of D-dimer as biomarker	D-dimer levels on admission predict disease outcome	Early risk stratification can guide therapeutic intensity	Clinical study [78]
Patients with COVID-19	Pathological analysis	Demonstrated endothelial cell infection and endotheliitis in COVID-19	Suggests potential benefit of therapies targeting endothelial protection	Clinical study [80]
N/A	Analysis of cytokine storm pathways	Reviewed signal pathways and treatment of cytokine storm	Specific recommendations for managing cytokine storm in COVID-19	Review [45]
